# Clinical Use of Aligners Associated with Nuvola^®^ OP System for Transverse Maxillary Deficiency: A Retrospective Study on 100 Patients

**DOI:** 10.3390/ijerph19095751

**Published:** 2022-05-09

**Authors:** Giovanna Perrotti, Alessandro Carrafiello, Ornella Rossi, Lorena Karanxha, Giulia Baccaglione, Massimo Del Fabbro

**Affiliations:** 1 Lake Como Institute, 22100 Como, Italy; giovanna.perrotti@lakecomoinstitute.com (G.P.); rossi.ornella@outlook.it (O.R.); 2 Private Practice, 42122 Reggio Emilia, Italy; alexcarrafiello@msn.com; 3 Department of Biomedical, Surgical and Dental Sciences, Università degli Studi di Milano, 20122 Milano, Italy; lorena.karanxha@unimi.it; 4 Private Practice, 20154 Milano, Italy; giulia.baccaglione@gmail.com; 5 IRCCS Orthopedic Institute Galeazzi, Via Riccardo Galeazzi 4, 20161 Milan, Italy

**Keywords:** aligners, maxillary palatal expansion, orthodontic treatment, superimposition, intraoral scan

## Abstract

Introduction: Aligners represent a common treatment for orthodontic patients thanks to their countless advantages including aesthetics, comfort, and oral hygiene maintenance; at the same time, they are associated with a reduced number of visits and a low incidence of complications. Although aligners have undergone considerable improvements over time, to date they have limitations in resolving the most serious malocclusions related to transverse maxillary deficiency. The aim of the present study was to retrospectively evaluate an orthodontic protocol (the Nuvola^®^ OP System) which associates a morpho-functional corrector (to be used for 30 min/day) with the aligner, allowing for the treatment of cases that would be difficult or unpredictable with aligners alone. Methods: Linear measurements were taken on STL files of 100 patients. In 77 patients between 18 and 55 years (mean 28.6 ± 16.2 (standard deviation) years), 3D superimpositions of the maxilla obtained from intraoral scans before and after treatment were performed in order to evaluate the degree of maxillary expansion. The normality of the data distribution was tested. The pre- and post-treatment data were compared using a paired t-test with a 5% significance level. After treatment, a questionnaire was proposed to assess patients’ degree of satisfaction and compliance. Results: A statistically significant difference (*p* < 0.05) for each distance evaluated was found. The maximum expansion was obtained at the first molar level (2.35 ± 1.64 mm). Of the subjects who completed the questionnaire, 96% were satisfied and 90% were able to perform the protocol without difficulty for the required duration. Conclusions: A significant expansion of the maxillary arch as well as a high degree of patient satisfaction and compliance were observed with the Nuvola^®^ OP System. Further studies are needed in order to clinically evaluate the relative contribution of the aligners and morpho-functional device to the obtained expansion.

## 1. Introduction

In recent years, an increasing number of adult patients requiring orthodontic treatment prefer “invisible” and comfortable aesthetic alternative to conventional fixed orthodontia [1,2], especially in the adult population with specific professional and socio-relational needs [3,4].

In 1946 Kesling proposed the sequential use of thermoformed plastic aligners to induce orthodontic movement in misaligned arches [5].

In 1997, Align Technology (Santa Clara, CA, USA) developed a therapy method with aligners through CAD/CAM technologies [6]. The growing demand for aligners has led to the improvement of treatment with them no longer being limited to simple cases of dental crowding, extending treatment to more complex malocclusions [7]. However, although treatment with aligners is considered a safe, aesthetic, and comfortable orthodontic procedure that allows a good hygienic maintenance for the patient [8,9], only a few studies [10,11] have focused on the real-world predictability of orthodontic movement, with an overall average accuracy between anticipated and achieved movements equal to only 41% [10].

In 2005 Lagrave’ re and Flores-Mir published a systematic review of the literature in which only two studies on the efficacy of Invisalign aligner therapy met their inclusion criteria. The authors concluded that an operator who decides to use them on patients must rely on his/her own clinical experience and on the opinions of the experts due to the limited scientific evidence [12]. Additionally, while most of the available studies are related to the Invisalign system, differences in material properties and aligner thickness, manufacturing process, model accuracy, and aligner margin position all have a different effect on the final performance of the orthodontic device [13,14,15,16]. Therefore, different results can be expected from different transparent aligner systems [17]. In combination with treatment with aligners alone, accelerators of orthodontic movement (Acceledent, OrthoPulse) have been proposed in order to achieve faster and more satisfactory outcomes; unfortunately, the results have not met expectations [18].

Other popular devices for the treatment of Class II malocclusions consist of prefabricated functional appliances (PFA). PFAs are removable orthodontic devices made of an elastomeric material that incorporates soft shields around the dental arches and reduces the pressure exerted by the perioral muscles. These devices aim to correct malocclusions by combining the characteristics of a functional appliance with those of an eruptive guide [19,20]. PFAs are soft, non-personalized, and often used in conjunction with myofunctional exercises [19,20]. They can only be worn part-time. PFAs have been available for several decades, and in recent years clinical studies have been published comparing their effects with more traditional functional appliances [21,22,23,24]. It has been reported that PFAs are able to correct various types of malocclusions in growing individuals [23,25,26,27] in order to induce dental movements in patients at the end of growth [22,23,25,28], reduce overbite [24,25,26,28], improve crowding issues [23,26], and correct Class II molars towards Class I [23,26]. Transversal effects of PFAs are less commonly reported. One study reported 1–1.5 mm of arch expansion after one year of use [29]. However, the skeletal effects reported in the literature remain highly controversial [22,25,26,27,28,30].

The aim of this study was to analyze a new system proposed by Nuvola^®^ (G.E.O. S.r.l., Rome, Italy), called the Nuvola^®^ OP System, which combines a functional appliance with therapy with modified transparent aligners using both linear measurements and superimposition of intraoral scans before and after treatment. The null hypothesis was that no transverse maxillary expansion would be achieved when using the Nuvola^®^ OP System.

## 2. Materials and Methods

This retrospective study evaluated a consecutive series of 100 patients who were treated in different clinics in Italy between November 2018 and July 2020 according to standard procedures. The cases were collected and provided by the company Biomax Spa, Vicenza, Italy, from a network of practitioners (orthodontics specialists) who had used the Nuvola^®^ OP System. Due to the retrospective nature of the study, no ethical review or approval was required. The inclusion criteria for the chart selection were adult patients with diagnosis of a transverse maxillary deficiency; intraoral scans taken before treatment and within three weeks after the termination of the treatment; no craniofacial abnormalities; no previous orthodontic treatment; and patients treated with the Nuvola^®^ OP system. Patients with lateral or anterior crossbite and patients affected by temporomandibular disorders (myogenic pain) were excluded. 

The required sample size was estimated by assuming a maxillary mean transverse expansion of at least 1 mm. From a preliminary evaluation of the transverse distance, a standard deviation of about 3 mm was estimated. Considering an Alpha of 5%, a Power of 80%, and a paired t-test, the minimum required sample size was 73. Assuming that 20–25% could be excluded for various reasons, due to possible heterogeneity of the data collected from different clinical practices it was decided to initially select 100 patients.

Pre- and post-treatment intraoral scans were performed. Linear measurements on the STL (“Standard Triangulation Language”) files of the upper arches before and after treatment were assessed. The myogenic pain at the temporomandibular joint was evaluated and excluded in all patients by stimulating clenching activity. Of the patients selected, 56% had posterior crossbite, no mandibular deviation due to maxillary constriction was reported, and 40%, 45%, and 8% of patients had malocclusion class 1a, 2a, and 3a, respectively, while 7% had asymmetric malocclusion.

After treatment, all patients were asked to fill in a questionnaire to investigate: 1. their degree of satisfaction, using a rating scale (very satisfied/satisfied/fairly satisfied/not satisfied); 2. their compliance (i.e., whether the patient was able to meticulously perform the protocol); 3. their frequency and duration of use; and 4. their comfort (whether their execution of the protocol caused any inconvenience). 

### 2.1. Nuvola^®^ OP System

The OP system is an orthodontic therapy system that combines specific aligners and a specially designed device (Freedom^TM^) based on polyurethane and other elements. Its purpose is to be bitten with the aligners in situ in order to add the classic orthodontic movement (based on light and constant forces) to an adaptation of the cranial sutures, the latter induced by the contraction of the masticatory muscles (e.g., pterygoid, masseter, suboccipital). This activity generates cyclical and strong forces (therefore not orthodontic), and is independent of the occlusion.

**Figure 1:**
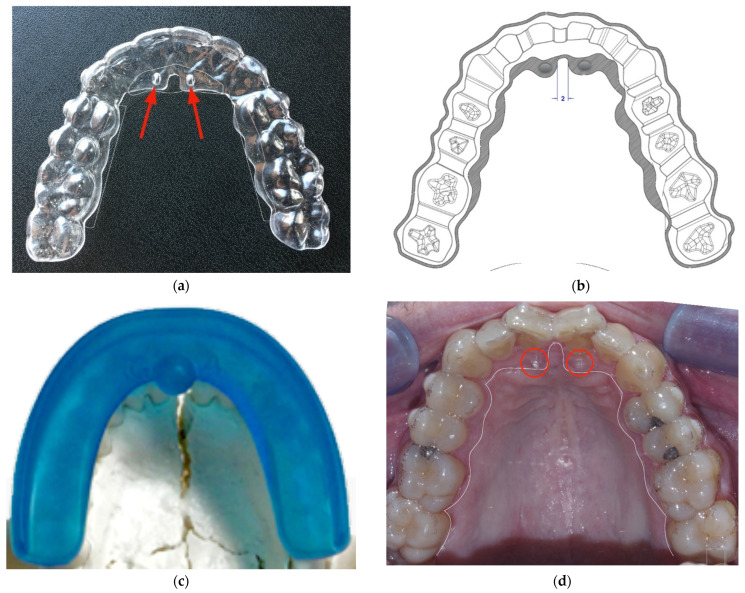
Composite figure showing the devices used in the present study: (**a**) in this panel, an aligner is shown with “lingual pins” indicated by the red arrows; (**b**) a drawing of the aligner; the gray areas represent the reinforced regions, which apply mastication forces to specific teeth groups; (**c**) here is shown the functional morpho-correcting Freedom^TM^ device (mounted on a skull model); and (**d**) a palatal view of a case with aligners in situ, with the pins indicated by red circles (the borders of the aligner are evidenced by a white line).

**Figure 2:**
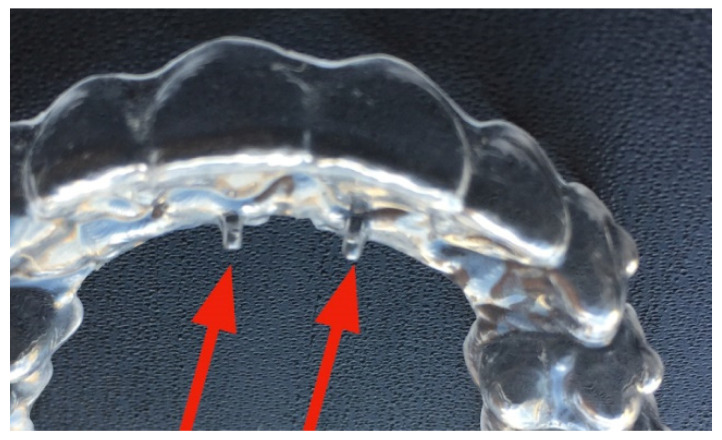
A detail of the aligner with the “lingual pins” in evidence, indicated by the red arrows.

Following the present protocol, the Freedom^TM^ should be clenched for 30 min a day with the aligners on, while the latter remain for the rest of the day. The aligners in turn have areas of reinforcement that stiffen specific dental groups, affecting certain palatal areas corresponding to the sutures present in the palate. The device includes the presence of two pins on the palatal side (called “lingual pins”), arranged in such a way as to orient the tongue towards the palatine spot, improving its functionality and making it act at the level of the premaxilla. Figure 1 and Figure 2 show the devices used in the present study.

### 2.2. Linear Measurements

To analyze the expansion movements, linear measurements were performed on the STL files of the upper arches pre- and post-treatment using Netfabb software (Autodesk, San Rafael, CA, USA) and on the overlapping of the pre- and post-treatment arches using the open-source software CloudCompare (version 2.11.1, www.cloudcompare.org and www.danielgm.net/cc/release accessed on 15 May 2021).

The following measurements were performed on both the pre-treatment and post-treatment data:

- D1 = distance between the palatine papilla and the mesial cusp of the sixth of the right hemi-arch;
- D2 = distance between the palatine papilla and the mesial cusp of the sixth of the left hemi-arch;
- D3 = distance between the canine-canine cusp;
- D4 = distance between the mesial cusp of the left and right first molar.

The measurements were registered by the same operator (G.P.), who had over five years of experience managing STL files. Before starting, a calibration was performed by measuring the same distances on a sample of ten patients. To obtain a measurement as reliable as possible for each distance considered, the same measurement was repeated six times in order to express the result as the mean ± SD. Only when the SD was less than 5% of the mean distance were the measurements performed on the included subjects.

### 2.3. Superimposition Assessment

Patients in whom scans of both arches and pre- and post-treatment occlusion bite were not available were excluded from the survey. In addition, patients in whom dental element was missing (in particular, the canines and/or first molars), were excluded from the superimposition analysis. 

The standard method for overlapping two STL files with CloudCompare was used as described in the User Manual. The color scale used in the present study ranges from blue (areas that have undergone less expansion) to red (areas that have undergone greater expansion), passing through the colours green, yellow and orange. Values from 0 to 4 were considered, and this interval was automatically divided by the software into eight symmetrical intervals.

### 2.4. Statistical Analysis

The Kolmogorov–Smirnov test of normality was used to analyze the type of data distribution. A paired Student’s *t*-test was then performed to assess whether there was a statistical difference between the pre- and post-treatment measurements. The significance level chosen for this analysis was 5% (α = 0.05). In addition, a linear regression analysis was performed for each distance between the post-treatment variation and the initial measurement to determine whether the extent of the variation was dependent on the pre-treatment value. A post hoc power analysis was performed considering the observed mean expansion at the first molar level at the end of treatment as the effect. A *p*-value < 0.05 was considered indicative of significant statistical difference.

## 3. Results

**Figure 3:**
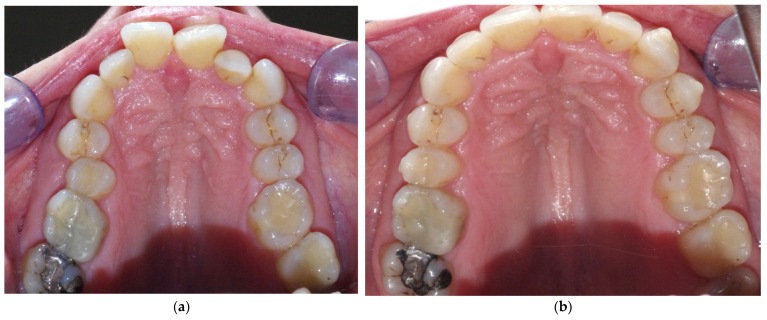
Upper occlusal view of a case treated with the Nuvola^®^ OP System (**a**) before treatment and (**b**) after treatment; the duration of the treatment was 18 months.

**Figure 4:**
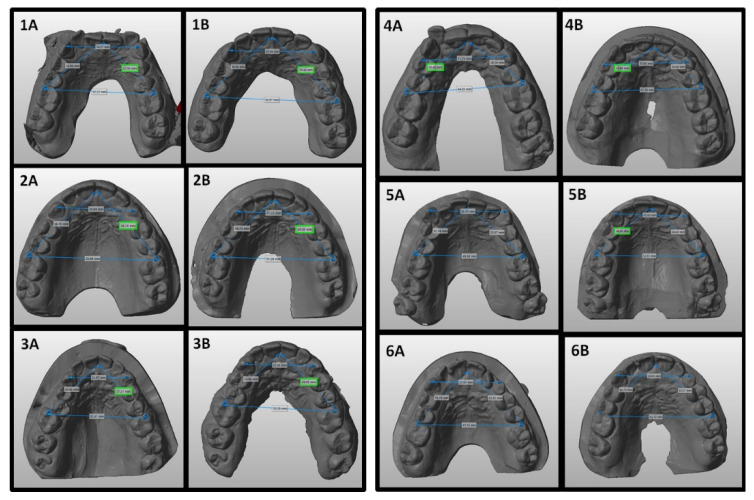
Linear measurements on STL files of six upper arches before treatment (**1A**–**6A**) and at the end of treatment (**1B**–**6B**).

The initial sample of 100 cases included 45 female and 55 male adult patients aged between 18 and 55 years (mean value 28.6 ± 16.2 years). Pre- and post-treatment measurements were taken in all cases; ultimately, only 77 cases were eligible for superimpositions as not all STL files were suitable due to poor quality that did not allow a correct analysis (fourteen cases) or to the absence of maxillary canines and/or first molars (nine cases). The orthodontic treatment used in this study had an average duration of between 18 and 26 months. Figure 3 shows the pre- and post-treatment upper occlusal view of a case. Figure 4 shows examples of pre- and post-treatment measurements from the intra-oral scans.

The results obtained from the statistical analysis show a significant difference (*p* < 0.05) for each measured distance. Post-treatment measures indicate a significant improvement over pre-treatment measures. The greatest expansion was obtained at the level of the first molar (mean value 2.35 ± 1.64 mm, 2.00 ± 1.65 mm for trimmed dental cast STLs files and 3.01 ± 1.60 mm for untrimmed dental cast STLs files).

A significant increase was found in the distance between the cusps of the canines (1.35 ± 1.74 mm), and in the distance between the palatine papilla and the mesial cusp of the sixth of the two hemi-arches, indicating an expansion of the arch (Table 1).

**Figure 5:**
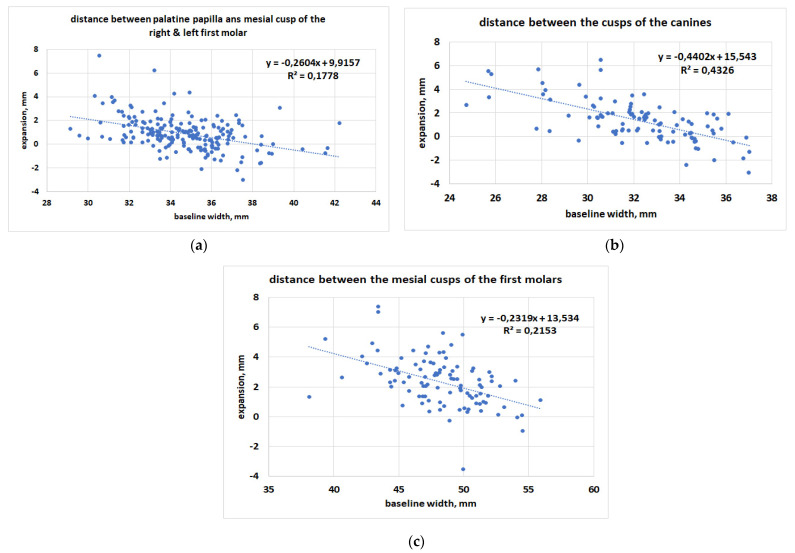
Linear regression analysis between the variation of a distance and its pre-treatment value: (**a**) distance between the palatine papilla and the mesial cusp of the first molar of the right + left half arch; (**b**) distance between the canine-canine cusp; (**c**) distance between the mesial cusps of the first molars.

From the means of the linear regression analysis, it can be seen that for all measured distances the variation in terms of expansion is greater for smaller arches (Figure 5a–c). In all cases the regression was significant, with *p* < 0.0001 (the slope was significantly different from zero).

**Figure 6:**
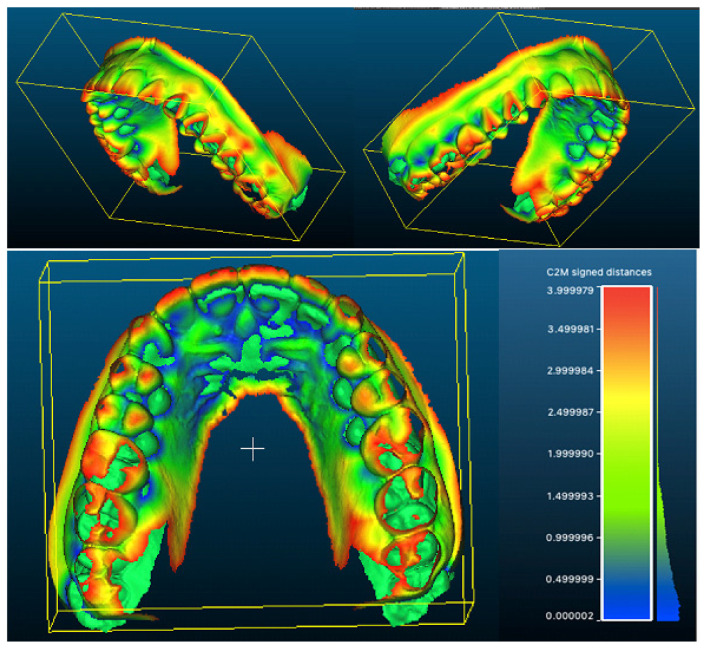
Superimposition of the upper arches with colorimetric scale (C2M = cloud-to-mesh signed distances).

A visual representation of the 3D superimposition of pre-treatment and post-treatment STL files is shown in Figure 6.

**Figure 7:**
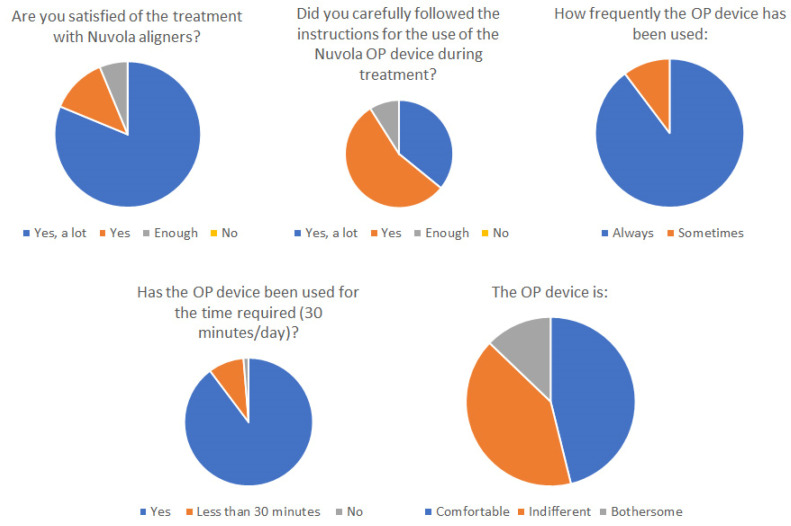
Results of the questionnaire distributed to patients after the end of treatment.

The questionnaire to assess compliance and satisfaction was completed by 77 out of 100 patients. The responses are summarized in Figure 7. In total, 96.1% of the subjects were satisfied with the treatment, and 90% declared that they strictly followed the protocol.

## 4. Discussion

The present study found that the Nuvola^®^ OP system (G.E.O. S.r.l., Rome, Italy) produces a statistically significant expansion of the maxillary arch in an adult sample. Specifically, it induces an increase in transverse diameters at the inter-canine and inter-molar and an increase of the length of the arch, allowing more space for the alignment of the teeth.

It might be hypothesized that this effect is partly due to the action of the reinforced shields of the morpho-corrector vestibular to the alveolar process, which should reduce the centripetal pressure provided by the perioral musculature, in particular by the buccinator muscle, and stimulate a transverse expansion of the maxillary arch, similar to what happens with the shields of Frankel’s device [31]. This action might allow great efficiency of the programmed expansion with the aligners and of the remodeling action of the tongue on the alveolar process. Of course, this should be confirmed with future controlled studies. Transverse expansion with aligners has been reported for the Invisalign^®^ system (Align Technology, San Jose, CA, USA). A recent study on 64 maxillary cases that used an analysis based on virtual models pre- and post-treatment reported expansion values at the canines, premolars, and first molar cuspid level, the latter being very similar to the present study [32]. However, in that study only medians with confidence intervals were reported instead of mean values. Another recent study using the Invisalign^®^ system in 28 adult patients with transverse discrepancy of 3–6 mm reported an expansion of 2.2 mm at the canine and 2.6 mm at the mesial cusp of first molar, very similar to the present findings [33], similarly worked with STL files of digital models from intraoral scans. 

The introduction of digital techniques represents a breakthrough in dentistry, and is becoming more and more popular for diagnosis, planning, treatment, and follow-up assessment. Techniques using the superimposition of images taken at different times represent a fundamental diagnostic and research tool for evaluating the effects of various orthodontic devices on the dental arches after treatment, as well as patients’ normal growth more generally [34]. The gold standard in orthodontics for superimpositions is latero-lateral teleradiography. However, this method has disadvantages such as difficulty in identifying reference points, radiological exposure, and the ability to evaluate only sagittal and vertical changes [35].

Modern diagnostic techniques such as cone-beam computed tomography (CBCT) could overcome the limitations of 2D investigations such as enlargement, distortion, and lack of three-dimensionality [36,37], although certainly not the radiation exposure, making CBCT suitable only for particular cases. Study models and intraoral scans represent a valuable alternative for performing superimpositions, avoiding biological risks related to radiological exposure. For the upper arch, palatal wrinkles can serve as a reference point for comparison before and after treatment [38]. Digital models can easily be aligned with any other landmarks to obtain the correct orientation in space [39].The Nuvola^®^ OP system is characterized by the additional use of intense and intermittent forces directed to the cranial bone bases deriving from the clenching of the Freedom^TM^ device, which are specially designed to stimulate skeletal adaptations of the skull base through specific myofunctional stimuli which the patient must perform independently for 30 min a day. This method does not require any metal elements, has minimal invasiveness, and is a technique where the operator’s manual skills have little effect. While analogous results are certainly achievable with traditional and surgical methods [40,41,42,43,44,45,46], have the drawbacks of greater invasiveness, higher costs (considering the various laboratory manufactured products), and greater variability depending on the manual skills of the various operators who take part in treatment. Furthermore, the “lingual pins” of the aligners seem to have a significant impact on the correction of tongue functionality. In fact, the pins remain active 24 h a day for the entire duration of the treatment, providing a constant corrective stimulus independent of the patient’s self-discipline.

One of the limitations of the present study is the lack of a control group represented by patients who used only the aligners or only the Morphofunctional corrector (Freedom) alone. Further studies are therefore necessary, for example, to compare the effect of the aligner on the dental arches with the effect of the aligner in addition to the morphofunctional corrector and with the effect of the morphofunctional corrector alone. Naturally, for a study of this type (case-control) it would be necessary to consider a sufficient number of pairs of patients with the same characteristics in terms of age, morphology, and initial clinical situation of the arches and who have been treated with one or the other technique. A further question that can be asked about functional devices, and therefore elastomeric morphocorrectors, especially when applied to patients in the growth phase, is what the direct effect of the device is compared to the effect due to natural growth. The prefabricated morphocorrector could stimulate further transverse development, overlapping that produced by natural growth, which can be a positive fact in the treatment of patients with crowding caused by a decrease in maxillary or mandibular transverse development.

A further limitation of this study, which, however, may be an indication for future developments, is the limited duration of the follow-up. Long-term post-treatment follow-ups with the Nuvola OP system could be extremely helpful in assessing treatment stability [29]. 

From the superimposition of the scans of the upper arch, it can be seen that the main variations were observed at the level of the posterior sectors, especially at the dental level and at the level of the incisor margins. Second-level investigations such as CBCT could help to more accurately assess skeletal changes. Furthermore, a current limitation of the superimpositions is that the results are only visual based on a colorimetric scale, and therefore do not allow the production of numerical data that would allow for more accurate statistical analysis.

## 5. Conclusions

- The Nuvola^®^ OP System is effective in obtaining dental expansion in the adult patient;
- Further studies are needed to evaluate skeletal changes using radiological 3D techniques such as CBCT;
- The system should be evaluated over the long term to assess the stability of changes;
- The separate contribution of aligners and the myofunctional device to the achieved expansion should be investigated.

## Figures and Tables

**Table 1:** Mean, standard deviation and significance of the difference between the values of the measured pre- and post-treatment distances. The asterisk indicates *p* < 0.05.

	Parameters	Pre-Operative (Mean ± SD)	Post-Operative (Mean ± SD)	Expansion (mean ± SD)	*p*-Value
**TOTAL STL (*n* = 100)**	D1-D1′(mm)	34.63 ± 2.22	35.48 ± 2.02	0.82 ± 1.32	*
	D2-D2′(mm)	34.69 ± 2.22	35.62 ± 2.09	0.93 ± 1.37	*
	D3-D3′(mm)	32.24 ± 2.60	33.59 ± 1.96	1.35 ± 1.74	*
	D4-D4′(mm)	48.19 ± 3.28	50.58 ± 2.91	2.35 ± 1.64	*
**Trimmed dental cast STL (*n* = 33)**	D1-D1′(mm)	34.84 ± 2.41	35.73 ± 2.32	1.01 ± 0.10	*
	D2-D2′(mm)	34.05 ± 2.42	35.90 ± 2.44	0.85 ± 1.14	*
	D3-D3′(mm)	32.36 ± 2.74	33.56 ± 1.90	1.20 ± 0.09	*
	D4-D4′(mm)	48.76 ± 3.30	50.76 ± 3.00	1.99 ± 0.09	*
**Untrimmed dental cast STL (*n* = 67)**	D1-D1′(mm)	34.58 ± 2.12	35.33 ± 1.84	0.75 ± 0.10	*
	D2-D2′(mm)	34.54 ± 2.08	35.49 ± 1.88	0.95 ± 0.11	*
	D3-D3′(mm)	32.13 ± 2.49	33.60 ± 1.99	1.47 ± 0.10	*
	D4-D4′(mm)	47.83 ± 3.21	50.84 ± 2.84	2.58 ± 0.08	*

## Data Availability

The data presented in this study are available upon request from the corresponding author.
